# Modeling conformational flexibility of kinases in inactive states

**DOI:** 10.1002/prot.25756

**Published:** 2019-06-17

**Authors:** Dominik Schwarz, Benjamin Merget, Charlotte Deane, Simone Fulle

**Affiliations:** ^1^ BioMed X Innovation Center Heidelberg Germany; ^2^ Department of Statistics University of Oxford Oxford UK

**Keywords:** conformational ensembles, DFG‐out, drug design, homology modeling, kinases

## Abstract

Kinase structures in the inactive “DFG‐out” state provide a wealth of druggable binding site variants. The conformational plasticity of this state can be mainly described by different conformations of binding site‐forming elements such as DFG motif, A‐loop, P‐loop, and αC‐helix. Compared to DFG‐in structures, DFG‐out structures are largely underrepresented in the Protein Data Bank (PDB). Thus, structure‐based drug design efforts for DFG‐out inhibitors may benefit from an efficient approach to generate an ensemble of DFG‐out structures. Accordingly, the presented modeling pipeline systematically generates homology models of kinases in several DFG‐out conformations based on a sophisticated creation of template structures that represent the major states of the flexible structural elements. Eighteen template classes were initially selected from all available kinase structures in the PDB and subsequently employed for modeling the entire kinome in different DFG‐out variants by fusing individual structural elements to multiple chimeric template structures. Molecular dynamics simulations revealed that conformational transitions between the different DFG‐out states generally do not occur within trajectories of a few hundred nanoseconds length. This underlines the benefits of the presented homology modeling pipeline to generate relevant conformations of “DFG‐out” kinase structures for subsequent in silico screening or binding site analysis studies.

## 1 INTRODUCTION

**Figure 1:**
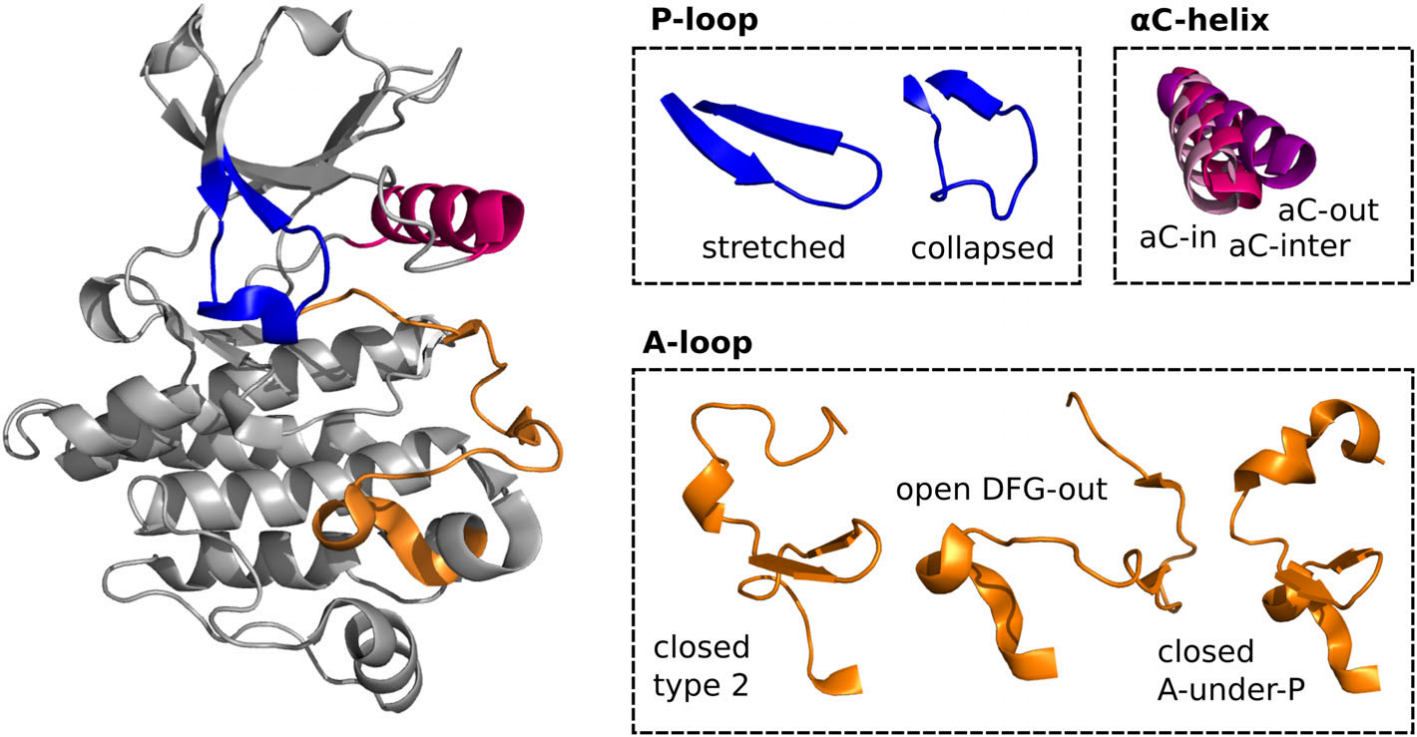
Structural elements of kinases that are considered for the generation of DFG‐out ensembles. P‐loop: stretched and collapsed; αC‐helix: αC{−in, −inter,‐out}; A‐loop: closed {type 2, A‐under‐P}, and open DFG‐out. Fusing these structural states results into 18 chimeric template structures for homology modeling

Protein kinases are key players of cellular signal transduction.^1^ Since misregulation of kinases or mutations occur in several diseases, such as cancer and inflammation, human kinases comprise a large group of potential drug targets.^2^ The catalytic domain of kinase structures has a bilobal architecture, with an N‐(terminal) lobe consisting of β‐strands and one α‐helix (called αC‐helix), and a C‐lobe consisting of mainly α‐helices. Connected by a flexible hinge region, the two lobes form a cleft with an evolutionarily conserved ATP‐binding pocket and the catalytic center.^3^ The C‐terminal domain consists of a flexible activation (A)‐loop, typically 20‐30 amino acids in length and marked by a conserved Asp‐Phe‐Gly (DFG) motif at the beginning of the A‐loop.^4^ The A‐loop can undergo large conformational changes, thereby controlling the catalytic activity and access to the substrate‐binding pocket. The conformational change is often accompanied by a specific orientation of the sidechains of the DFG motif, which leads to binding of different classes of inhibitors. In the catalytically active “DFG‐in” state, two Mg^2+^‐ions are positioned by the DFG aspartate and the catalytic loop, which coordinate the transfer of ATP phosphate groups.^5^ Compounds that preferentially bind to the DFG‐in conformation are called type I inhibitors. In turn, in the inactive “DFG‐out” conformation, the side chains of Asp and Phe of the DFG motif are flipped compared to the DFG‐in state, leading the Asp pointing away from the binding site. This prevents Mg^2+^‐coordination and, hence, catalytic activity.^3^ The DFG‐out conformation furthermore creates a hydrophobic pocket adjacent to the ATP binding site that can be targeted by the so‐called type II inhibitors. Another important structural element‐determining catalytic activity is the position of the αC‐helix, which consists of a conserved Glu residue and forms in the active state a crucial salt bridge with Lys of the N‐lobe β3‐strand but is usually moved outward in the inactive (DFG‐out) state (Figure 1).^5^, ^6^ Overall, there are a plethora of inactive conformations with varying A‐loop, DFG, and αC‐helix orientations. Furthermore, the P‐loop (also called G‐loop) is also highly flexible and can stack down onto the ligand, thereby creating a more buried cavity and stabilizing interactions with the ligand. A detailed analysis and comprehensive classification of the sampled conformation of kinases in the Protein Data Bank (PDB) can be found in a study by Möbitz.^5^

Both states, DFG‐in and DFG‐out, have been successfully employed for the design of approved kinase inhibitors^7^, ^8^ and have promises and challenges for the design of novel kinase inhibitors.^9^, ^10^ For instance, due to the high structural conservation of the active site, developing selective kinase inhibitors might be essential to reduce undesired off‐target activities.^11^, ^12^, ^13^ Structure‐based design efforts rely on structures in atomic detail and benefit from the presence of structural ensembles. Of all kinase structures available in the PDB, the “DFG‐out” conformation covers only ~15% of all typical human kinases, which is much lower than the ~45% covered in the DFG‐in state. This hinders classical homology modeling based on single template structures and already resulted in several attempts to model the DFG‐out states via more sophisticated methods. For instance, the DOLPHIN (deletion‐of‐loop Asp‐Phe‐Gly‐in) method^14^ removes the DFG‐Phe and four adjacent residues from DFG‐in structures in order to resemble the hydrophobic pocket occupied by type II inhibitors. The activation‐loop remodeling method (ALRM) method^15^ accounts for variability in the A‐loop and an N‐lobe rotation, while the DFGmodel approach^16^ generates multiple models for a single kinase based on a selection of representative template structures considering mainly the relative positions of the N/C‐lobes. However, none of these three approaches considers the structural diversity of all structural elements characterizing inactive (“DFG‐out”) structures. On the other side, large‐scale approaches, such as employing all available kinase catalytic domain structures to construct models of the human tyrosine kinase family,^17^ are very computationally demanding and may only shift the decision which structures to use for subsequent analysis.

Here, we describe a systematic generation of kinase structures in different DFG‐out states by generating multiple possible combinations of the A‐loop, P‐loop, and αC‐helix. Thus, the presented homology modeling approach accounts for the flexibility of the inactive state of kinases and generates a representative subset of “DFG‐out” conformations for binding site analysis or screening studies.

## 2 MATERIALS AND METHODS

### 2.1 Structural classification

In order to group the relevant “DFG‐out” conformations (Figure 1), all available PDB structures of human kinase domains (dated 08/09/2016) were classified based on geometrical features as described below. All residue IDs refer to the numbering of the PKACα structure with the PDB code 1ATP.

#### 2.1.1 DFG classification

The conformation of the DFG motif was initially determined to select only “DFG‐out” structures as templates of the kinase N‐lobes. The employed DFG classification is based on cross products of vectors of four atoms of the DFG motif as described by Xu et al.^15^ All DFG‐out structures in the PDB were further characterized based on structural variations in A‐loop, P‐loop, and αC‐helix.

#### 2.1.2 A‐loop classification

Kinase structures in the PDB were previously clustered by Möbitz into 14 major conformations based on pseudotorsional angles between four consecutive C_α_ atoms around the DFG motif.^5^ Three main clusters of A‐loop conformations for “DFG‐out” structures were initially defined based on visual inspection and called in analogy to Möbitz “closed type 2,” “open DFG‐out,” and “closed A‐under‐P” throughout the article. They were identified by employing pseudotorsional angles *xi*_DFG{−1, D}_ and *xi*_DFG{F, G}_ (already used by Möbitz^5^) and a C_α_‐C_α_ distance criterion between a residue in proximity to the His–Arg–Asp (HRD) motif in the upper right part of the C‐lobe (ie, HRD_−4_; ID 160) and a residue in the A‐loop (ie, DFG_+3_; ID 189) in order to obtain more homogeneous clusters (Table S1).

*P‐loop classification* is based on the evaluation of four structural features: two backbone dihedrals of the residues just before (ID 49; *psi*_G‐motif‐1_) and after (ID 56; *psi*_G‐motif + 1_) the GxGxPhiG motif, a pseudotorsional angle between the C_α_ atoms of the four residues following *Phi* (IDs 55‐58; *xi*_G‐motif{+1, +2}_), and a C_α_‐C_α_ distance between the *Phi* residue (ID 54) and HRD_+4_ (ID 170). If at least three of these conditions are fulfilled (see Table S2), the P‐loop is classified as either “collapsed” or “stretched.” The four features were extracted from a feature importance analysis, employing a random forest classifier trained on manual P‐loop class annotations (see Figure S4 for further details of the classifier development).

*αC‐helix classification* is done following the rules described by Brooijmans et al^6^ that employs the minimal distance between the catalytic Lys (ID 72, atom NZ) and αC‐helix's Glu (ID 91, OE1, or OE2) to differentiate between αC‐in (*d* ≤ 4 Å) and αC‐out (d ≥ 8.5 Å) conformations. For distances in between, the αC‐helix's Glu dihedral *chi*_*+*1_ is considered (ie, αC‐inter: if angle ≤100°; αC‐in: otherwise).

### 2.2 Homology modeling

Homology modeling was performed with the YASARA program,^18^ employing pre‐prepared template structures and alignments as well as the following parameters: the number of templates to use: 1; the number of ambiguous alignments to consider per template: 1; the number of samples to try per loop: 25; and the maximum number of unaligned terminal residues to model: 10.

#### 2.2.1 Template construction

Template structures consisted of (rigid) C‐lobes of a corresponding “DFG‐in” structure (without the A‐loop), an N‐lobe of one of the six selected N‐lobe structures, and an A‐loop of one of the three selected A‐loop structures (Tables 1 and 2). Full kinase domains of this “N‐lobe” and “A‐loop” structure representatives were structurally aligned to the C‐lobe of “DFG‐in” structures by only considering the C‐lobe residues (without the A‐loop). Then, all residues except for the desired ones of the respective structure were deleted and the remaining structural elements joined into one “chimeric template structure.” Finally, a short energy minimization was performed to remove steric clashes.

**Table 1:** N‐lobe templates for DFG‐out structures with certain P‐loop/αC‐helix combinations

Structural class		Number of		Selected structure	
P‐loop	αC‐helix	PDB structures	Unique kinases	PDB code	Kinase
Collapsed	αC‐in	33	6	4QQ5; chain A	FGFR4 (TK)
	αC‐inter	4	3	2G2H; chain B	ABL1 (TK)
	αC‐out	5	4	5HX6; chain A	RIPK1 (TKL)
Stretched	αC‐in	246	41	3VHK; chain A	KDR (TK)
	αC‐inter	39	23	4PMM; chain A	TRKA (TK)
	αC‐out	51	15	2W5B; chain A	NEK2 (Other)

**Table 2:** A‐loop templates for DFG‐out structures with certain A‐loop conformations

Structural class	Number of		Selected structure	
A‐loop	PDB structures	Unique kinases	PDB code	Kinase
Closed type 2	37	10	3V5Q; chain A	TRKC (TK)
Open DFG‐out	55	16	2HZI; chain A	ABL1 (TK)
Closed A‐under‐P	88	17	3BEA; chain A	FMS (TK)

#### 2.2.2 Target‐to‐template alignment

The alignment provided in Reference 5 by Möbitz is a manually curated multiple sequence alignment of nearly 500 kinases. The sequence parts of the chimeric template structures were aligned to the corresponding sequence parts of the same kinase in the Möbitz alignment using the pairwise2.align.globalmc alignment function in Biopython, joined into one sequence, and finally employed as template sequence. The canonical catalytic kinase domain sequence from UniProt was employed as target sequence in the modeling step and accordingly also aligned to the alignment (with the exception of sequences of the kinases MASTL, SgK494, NEK10, and MNK1 whose UniProt sequences were either lacking important sequence parts or contained unwanted insertions). Hence, their target sequences were taken from References 5 and 19. A list of UniProt IDs can be found on http://www.kinhub.org/kinases.html.

#### 2.2.3 Input structure selection

“DFG‐in” input structures (for the C‐lobe templates) were taken from an in‐house “selected set” of kinase structures (ie, updated version of the version described in Reference 13) that consists of manually selected high‐quality DFG‐in structures per kinase. The following criteria were considered for the selection of the “selected set”: high resolution, DFG state (in/out), and structural completeness (especially in terms of having resolved A‐ and P‐loops), and favoring structures bound to ATP or ATP analogues. If a kinase was not present in the “selected set,” a homology model was generated employing the closest structure in the “selected set” as a template.

#### 2.2.4 Template structures

A total of 18 chimeric template structures were generated that represent the structural variation of the DFG‐out state and consist of two major P‐loop (ie, collapsed and stretched), three αC‐helices (ie, αC‐{in, inter, out}), and three A‐loop conformations (ie, closed type 2, open DFG‐out, and closed A‐under‐P). The selected N‐lobe and A‐loop template structures as well as one exemplary set of generated homology models (ie, for ABL1) can be obtained from https://github.com/Team-SKI/Publications.

### 2.3 Molecular dynamics simulations

#### 2.3.1 Structure preparation and molecular dynamics parameters

ABL1 homology models of types 8, 12, and 16 were protonated, parameterized with the ff14SB force field, charge neutralized with Na^+^ ions, and solvated with TIP3P water using the tLeAP module of AmberTools16.^20^ All simulations were carried out with NAMD,^21^ using 2 fs simulation time steps and the SHAKE^22^ algorithm to restrain all bonds to hydrogen atoms. The nonbonded energy calculation cut‐off distance was set to 12 Å, switching distance to 10 Å, and pair list distance to 13.5 Å with 10 steps per cycle. Electrostatic interactions were calculated with particle mesh Ewald (PME) method^23^ and temperature regulated with Langevin dynamics.

#### 2.3.2 Equilibration

10 000 steps of minimization were followed by a 1 ns equilibration in the NVT ensemble which heats from 100 K to 300 K by increasing the temperature by 1 K every 2.5 ps. Initial 0.5 kcal/(mol A^2^) constraints were gradually turned off after 100 ps until 500 ps, by 0.1% every 4 ps, ending the equilibration with another 500 ps of unconstrained simulation. 200 ns production runs were carried out in the NPT ensemble at 1.01325 bar with the Langevin piston Nosé‐Hoover barostat.^24^, ^25^

#### 2.3.3 Trajectory analysis

Coordinates were taken at regular intervals of 10 ps during the simulation. To assess the structural variability of the obtained homology models, backbone root‐mean‐square deviations (RMSDs) to the energy‐minimized starting structures were calculated (Figures S2 and S3). VMD 1.9.2 was used for aligning the trajectories to the corresponding starting structures and calculating the RMSD values. The statistical framework R was used for analysis and visualization.

### 2.4 Kinome tree plots

All kinome tree plots were generated via KinMap (http://www.kinhub.org/kinmap/).^26^

## 3 RESULTS

### 3.1 Structural classification

The generation of an “DFG‐out” ensemble is based on the usage of selected structures comprising the major different states of flexible structural elements of the kinase domain (Figure 1) and a subsequent fusion of these elements to multiple chimeric template structures. The initially selected structures were obtained by classifying all “DFG‐out” structures in the PDB with respect to the possible configurations of the A‐loop, P‐loop, and αC‐helix (see the Materials and Methods section). The analysis revealed that three major structural variants of the A‐loop, two of the P‐loop, and three of the αC‐helix exist (Tables 1 and 2).

Furthermore, since seven variants occurred in at least six out of the eight kinase groups, and the remaining variant (A‐loop: closed type 2) in three, we assumed that they are all distributed across the kinome (Data S1, Figure S1). Noteworthily, a kinase profiling study of presumable type II (DFG‐out) inhibitors revealed that more than 200 kinases, covering all branches of the kinome, were targeted by this set of inhibitors.^9^ This suggests that DFG‐out conformations are commonly sampled in the majority of kinases, and thus, seem not to be a unique feature of a particular subset of kinases. Thus, we assumed in the modeling pipeline that all kinases are able to adopt all possible combinations of the A‐loop, P‐loop, and αC‐helix.

Compared with the DFG‐in state, the N‐lobe in the DFG‐out state is rotating relative to the C‐lobe. Hence, we aimed for identifying one template each for the N‐lobe and C‐lobe as well as for the A‐loop. The C‐lobe (excluding the A‐loop) can be considered to be rigid (ie, it is not affected by the DFG conformation) and was taken from either a manually selected high‐quality DFG‐in structure of the respective kinase or a generated homology model (see the Materials and Methods section for more details as well as Reference 13). The N‐lobe can sample two major P‐loop and three major αC‐helix conformations (ie, P‐loop: collapsed and stretched; αC‐helix: αC‐{in, inter, out}), resulting in six possible combinations. The αC‐helix can be classified depending on whether a salt bridge can be formed with the catalytic Lys of the N‐lobe (αC‐in; present in 80% of active but only in 36% of inactive conformations^5^) or not, either due to a rotation of the αC‐helix (αC‐out) or despite no larger conformational changes of the αC‐helix (αC‐inter). The final selection of structures for the N‐lobe part is listed in Table 1 and only includes the structures with a “DFG‐out” motif, which passed geometrical criteria for these classes (see the Materials and Methods section), and contained no ambiguous or missing residues in the N‐lobe.

The A‐loop samples three respective clusters in DFG‐out structures (ie, closed type 2, open DFG‐out, and closed A‐under‐P). “Closed type 2” is the classical DFG‐out conformation for type II inhibitors, where the side chains of Asp and Phe of the DFG motif are flipped compared with the DFG‐in state, and the A‐loop is in a closed folded conformation. In contrast, the A‐loop is, similar to DFG‐in structures, in an unfolded conformation in the “open DFG‐out” conformation that opens up the active site and is compatible with binding of type I inhibitors. “Closed A‐under‐P” conformations contain again A‐loops in a closed conformation, but which form stabilizing stacking interactions with the P‐loop. Please note that while Möbitz^5^ differentiated further between characteristic A‐under‐P conformations, we assigned them all to one structural class. An extended A‐loop classifier (see the Materials and Methods section) was employed to cluster all kinase structures in the DFG‐out state into these A‐loop conformational classes, whereas the final selection for the A‐loop templates also considered the completeness of the loop (Table 2).

### 3.2 Homology modeling pipeline

**Figure 2:**
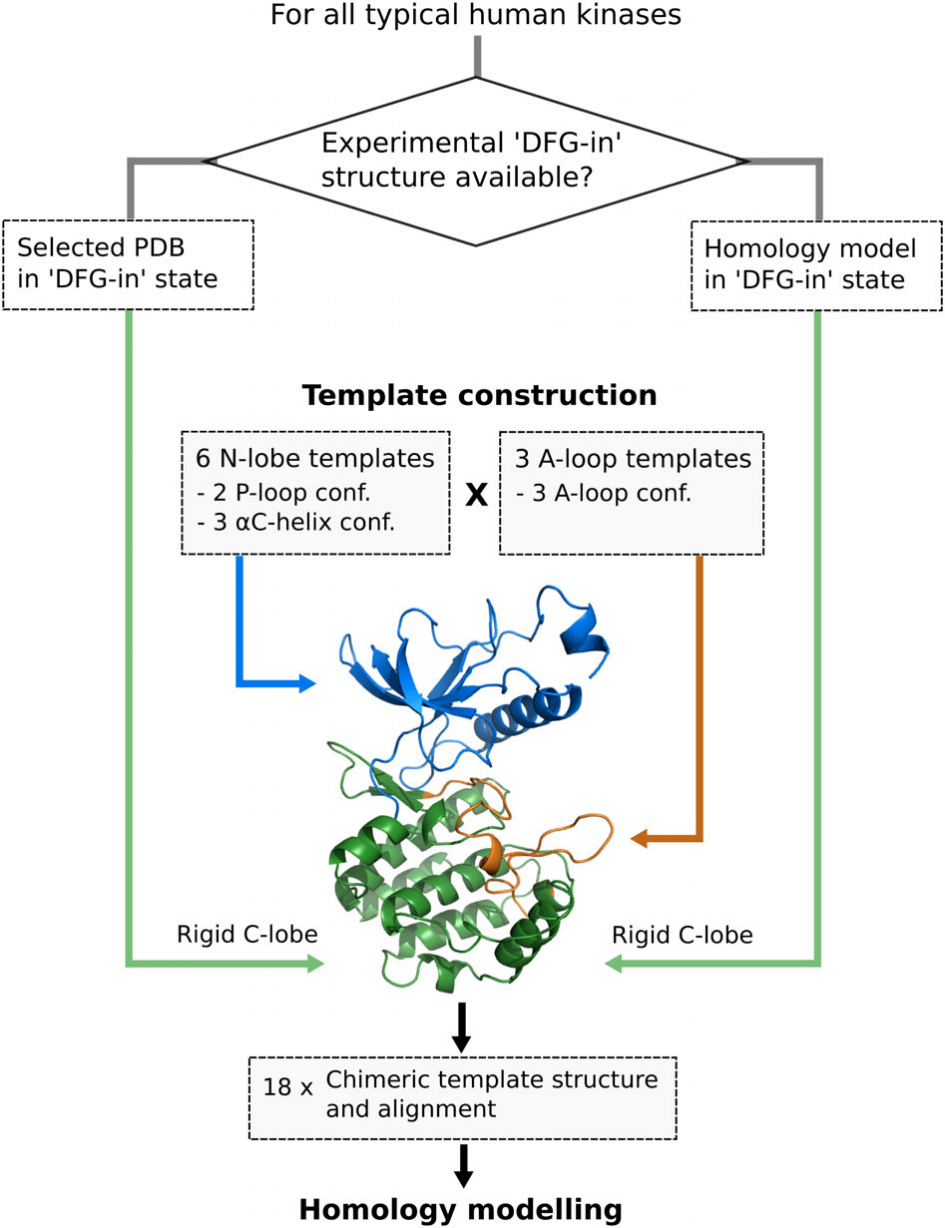
Scheme of homology modeling pipeline for modeling the entire kinome in different DFG‐out conformations. Template structures are constituted by a C‐lobe of a corresponding DFG‐in structure (green), an N‐lobe of one of the six selected N‐lobe template structures (blue), and an A‐loop of one of the three selected A‐loop templates (orange), resulting into 18 chimeric template structures per kinase [Color figure can be viewed at http://wileyonlinelibrary.com]

Examples of the explicitly considered flexible structural elements of kinases as well as the homology modeling pipeline are displayed in Figures 1 and 2. The selected N‐lobe (ie, αC‐helix and P‐loop) and A‐loop variants are used in all possible combinations together with one C‐lobe structure, forming a total of 18 chimeric templates. A target‐to‐template alignment was created by using the respective sequences of the target kinase and template segments of a manually curated multiple sequence alignment provided in Reference 5 (see the Materials and Methods section).

#### 3.2.1 Homology modeling of kinases in the DFG‐out state

**Figure 3:**
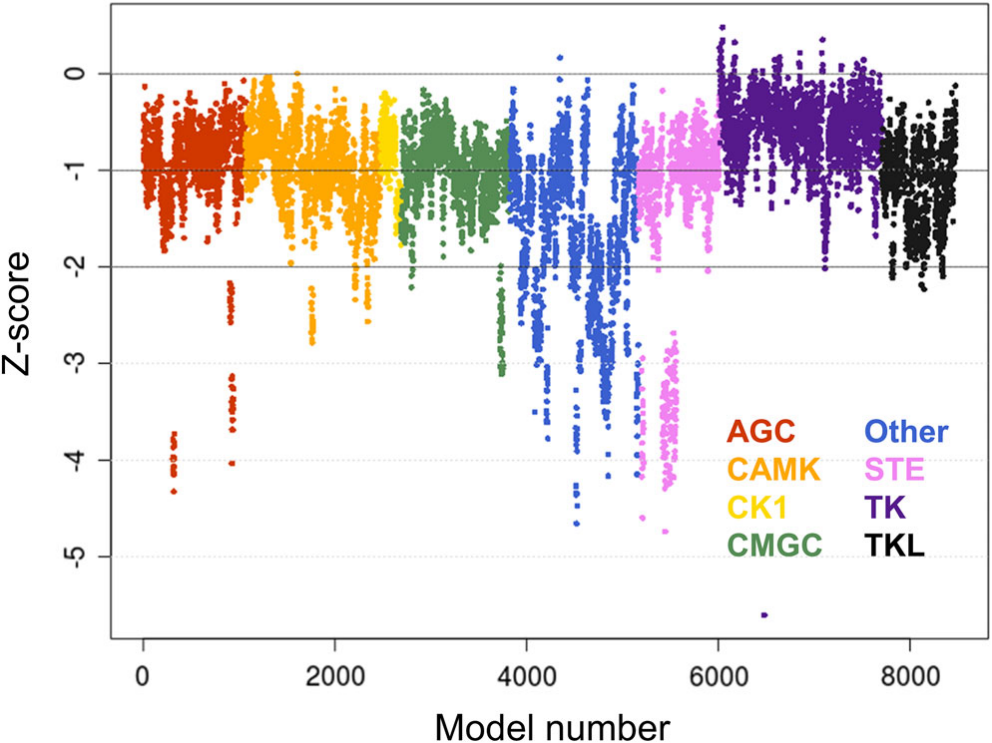
Distribution of *Z*‐scores obtained for the generated homology models. Each kinase is represented by 18 models and color coded according to the respective kinase group (ie, AGC, red; CAMK, orange; CK1, yellow; CMGC, green; Other, blue; STE, purple; TK, violet; and TKL, black) [Color figure can be viewed at http://wileyonlinelibrary.com]

Almost all typical human kinases could successfully be modeled in different structural states. *Z*‐scores, which estimate the overall quality of a model, indicate that all groups beside the “Other” group have a good quality with respect to dihedrals and packing parameters (Table 3). The best results were obtained for the TK group (median − 0.54 ± 0.32), the lowest *Z*‐scores for the “Other” group (−1.58 ± 0.89), while the remaining six groups have median values around −1. Individual *Z*‐scores of all obtained homology models, ordered by groups, are depicted in Figure 3. Models of the CK1, TK, and TKL groups are in general of good quality as only a few models obtained *Z*‐scores below −2. The AGC, CAMK, CMGC, and STE groups possess individual outlier kinases that score below −2 (note that the vertical series of adjacent data points usually display the models of a single target kinase). Visual inspection of randomly selected structures did not reveal any conspicuous features of these structures, such as unexpected folds or clashes. The distribution of *Z*‐scores of the “Other” group is much more scattered with scores ranging from −4.5 to about 0, presumably displaying the heterogeneity of this kinase group.

**Table 3:** Summary of generated homology models per kinase group

Kinase group	*Z*‐score^a^	Number of missing kinases
AGC	−0.87 ± 0.33	4/63
CAMK	−0.95 ± 0.39	4/82
CK1	−0.80 ± 0.33	0/12
CMGC	−1.00 ± 0.34	0/63
Other	−1.58 ± 0.89	8/82
STE	−1.07 ± 0.41	1/48
TK	−0.54 ± 0.32	1/94
TKL	−1.06 ± 0.44	1/43

a Median and median absolute deviation of *Z*‐scores for each kinase group, as calculated by the YASARA program. *Z*‐scores describe how many standard deviations the model quality differs from typical high‐resolution X‐ray structures. Scores above 0 are considered to be “optimal,” between 0 and −1 “good,” between −1 and −2 “satisfactory,” between −2 and −3 “poor,” and below −3 “bad.”

Out of the available PDB structures, ~160 structures could be assigned classes for all three flexible elements and, thus, provide a valuable source for comparison with the corresponding homology models. Matching PDB structures were found for 13 of the 18 model types. Visual comparison of these structural pairs let to a manual setting of a backbone RMSD cut‐off of 0.7 Å to declare matching pairs as structurally similar or different. Structural pairs below this threshold generally displayed not only a “classification match” (according to A‐loop, P‐loop, and αC‐helix classifiers based on angle and distance criteria) but also a “visual match” (similar shapes of the flexible features). Based on this threshold, at least one generated homology model was very similar to 10 of the 18 model types of experimental structures of which most were also, in general, similar in the course of respective molecular dynamics (MD) trajectories (Table 4). No matching PDB structures could be found for 5 model types consisting all of collapsed P‐loop conformations. According to the analysis of available PDB structures, this P‐loop conformation might prefer an A‐loop in the open DFG‐out state compared with closed type 2 and A‐under‐P conformations. Overall, the structural comparisons with existing PDB structures underline the value of the presented ensemble generation pipeline to generate scarcely populated conformations for further analysis and calculations.

**Table 4:** RMSD values of matching PDB structures and homology models

HM model type	A‐loop	P‐loop	αC‐helix	Matching PDB structures a	Mean RMSD	Minimal RMSD b
HM 1	Closed type 2	Collapsed	αC‐in	17	0.59 ± 0.05	0.5 (ABL1, 2E2B_B)
HM 2	Open DFG‐out	Collapsed	αC‐in	7	0.53 ± 0.05	0.5 (ABL1, 2HZI_A)
HM 3	Closed A‐under‐P	Collapsed	αC‐in	0	‐	‐
HM 4	Closed type 2	Collapsed	αC‐inter	0	‐	‐
HM 5	Open DFG‐out	Collapsed	αC‐inter	3	0.51 ± 0.06	0.5 (ABL1, 2G2H_B)
HM 6	Closed A‐under‐P	Collapsed	αC‐inter	0	‐	‐
HM 7	Closed type 2	Collapsed	αC‐out	0	‐	‐
HM 8	Open DFG‐out	Collapsed	αC‐out	3	1.28 ± 0.32	1.1 (LIMK2, 4TPT_B)
HM 9	Closed A‐under‐P	Collapsed	αC‐out	0	‐	‐
HM 10	Closed type 2	Stretched	αC‐in	11	1.00 ± 0.19	0.6 (FGFR4, 4TYJ_A)
HM 11	Open DFG‐out	Stretched	αC‐in	14	0.73 ± 0.17	0.5 (ABL1, 3UE4_B)
HM 12	Closed A‐under‐P	Stretched	αC‐in	69	0.70 ± 0.18	0.4 (KDR, 3VO3_A)
HM 13	Closed type 2	Stretched	αC‐inter	2	1.18 ± 0.02	1.2 (AMPKa2, 2YZA_A)
HM 14	Open DFG‐out	Stretched	αC‐inter	6	0.70 ± 0.22	0.5 (MAP3K5, 2CLQ_A)
HM 15	Closed A‐under‐P	Stretched	αC‐inter	4	0.79 ± 0.09	0.7 (BRAF, 4R5Y_B
HM 16	Closed type 2	Stretched	αC‐out	2	1.00 ± 0.00	1.0 (CDK6, 1G3N_A)
HM 17	Open DFG‐out	Stretched	αC‐out	9	1.23 ± 0.64	0.5 (ABL1, 4YC8_B)
HM 18	Closed A‐under‐P	Stretched	αC‐out	11	0.89 ± 0.09	0.7 (LOK, 4AOT_B)

a RMSD cut‐off for declaring a matching PDB structure/homology model pair as structurally similar was set to 0.7 Å.

b Corresponding kinase is given in brackets.

### 3.3 Probing structural variability/invariability with MD simulations

**Figure 4:**
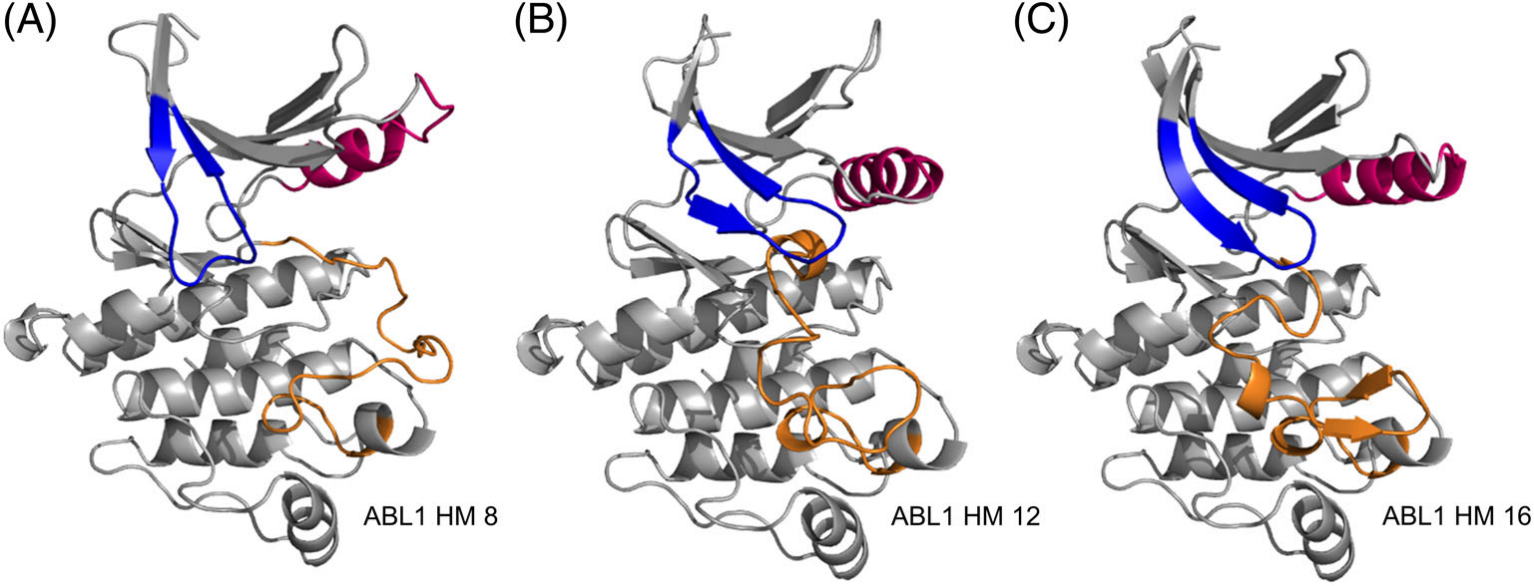
Homology models of ABL1 that were used as starting structures of molecular dynamics simulations. P‐loops are colored in blue, αC‐helices in pink, and A‐loops in orange. The A‐loop conformation in model type HM 8 is “open DFG‐out,” in HM 12 “closed A‐under‐P,” and in HM 18 “closed type 2” [Color figure can be viewed at http://wileyonlinelibrary.com]

MD simulations of 200 ns length were run to assess the general structural variability of the generated homology models. ABL1 homology models of types 8, 12, and 16 were selected to include all three A‐loop conformations (Figure 4; Table 5). The variability of the two P‐loop conformations (“stretched” and “collapsed”) was also assessed as well as the two most different states of the αC‐helix (“αC‐in” and “αC‐out”).

**Table 5:** Homology models chosen as starting structures for MD simulations

Homology model	A‐loop class	P‐loop class	αC‐helix class	Mean RMSD
ABL1—HM 8	Open DFG‐out	Collapsed	αC‐out	2.64 ± 0.55
ABL1—HM 12	Closed A‐under‐P	Stretched	αC‐in	2.26 ± 0.25
ABL1—HM 16	Closed type 2	Stretched	αC‐out	3.19 ± 0.34

The structural classifications refer to the chimeric template that was used to build the respective homology model. Mean backbone RMSD values were calculated over MD trajectories of 200 ns length with respect to their energy‐minimized starting structure.

Visual inspection of the trajectories together with backbone RMSD calculations with respect to their energy‐minimized starting structures (Table 5; Figure S2) indicates that ABL1 HM 12 is overall stable (ie, invariable) along the trajectory (mean RMSD: 2.26 ± 0.25), while small conformational changes occur during the HM 8 (mean RMSD: 2.64 ± 0.55) and HM 16 (mean RMSD: 3.19 ± 0.34) trajectories due to movements of the αC‐helix and its connecting loop to the N‐lobe's β‐sheet. The conformational change sampled in the HM 8 trajectory might result from a suboptimal choice of the N‐lobe template structure (5HX6; chain A), which possesses an unusually short αC‐helix but was the only template structure matching this particular combination of P‐loop and αC‐helix at the time of the present study. Thus, another N‐lobe template with a “collapsed” P‐loop and an αC‐helix in an “αC‐out” configuration might improve the quality of future homology modeling calculations. Apart from a turn formation of the αC‐helix, the model structure is stable along the trajectory and does not undergo noteworthy conformational changes. In the case of the ABL1 HM 16 trajectory, a slight anticlockwise rotation of the N‐lobe and subsequent electrostatic interactions between the connecting loop between αC‐helix and β‐sheet and the C‐lobe occurs. After formation of these interactions, the RMSD increases very slowly throughout the simulation, and visual inspection shows no major structural changes.

The conformational changes sampled in the three MD trajectories were mainly related to movement of the αC‐helix or its connecting elements, which is in line with the general mobile nature of the αC‐helix.^1^, ^27^ As expected, larger conformational changes (such as necessary for the A‐loop) did not occur in any of the three MD trajectories, as they occur on the microsecond to millisecond timescale.^28^ This underlines the benefit of the presented modeling pipeline to generate an ensemble of inactive (“DFG‐out”) structures as well as the overall structural invariability of the generated homology models in the course of MD simulations of a few hundreds of nanoseconds length. An additional comparison of all three simulation trajectories against all other homology model types further supports this conclusion as, besides again the αC‐helix configuration, no conformational transitions occur between the structural classes (Figure S3).

### 3.4 Testing model ensembles for docking studies

Applications of the generated DFG‐out ensembles are manifold, including their application for in silico screening and binding site analysis studies. To test whether our model ensembles are suited for docking studies, we selected FDA‐approved type II inhibitors of the two kinases ABL1 and KDR (five cases in total; Table 6) and docked them into the generated DFG‐out ensembles for those two kinases. Encouragingly, docking poses with good agreement to the crystal structure (ie, RMSD <2.2 Å) were found within the top five docking poses for four out of the five cases (Tables S4 and S5), and at least in the case of the ABL1 structure many of the corresponding receptor models have the same structural features /classification as the respective crystal structure (see Data S1 for more details).

**Table 6:** Small docking set of FDA approved type II inhibitors

Kinase	PDB code	Docked compounds
ABL1	2HYY, 3CS9, 3OXZ a	Imatinib, nilotinib, ponatinib
KDR	3WZE, 4AG8 b	Sorafenib, axitinib

a All three ABL1 crystal structures are classified as A‐loop: “closed type 2,” P‐loop: “collapsed,” and αC‐helix: “αC‐in” (which is equivalent to HM 1).

b All two KDR crystal structures are classified as A‐loop: “closed A‐under‐P,” P‐loop: “stretched,” and αC‐helix: “αC‐in” (which is equivalent to HM 12).

For the three ABL1 cases, the respective crystal structures are all classified as A‐loop: “closed type 2,” P‐loop: “collapsed,” and αC‐helix: “αC‐in” (which is equivalent to HM 1). For two of the respective docking cases (imatinib and nilotinib), a good docking pose and the correct model class were found within the top five docking poses, and for all three cases all receptor models of the top poses have at least a closed A‐loop conformation. For the two KDR cases, the respective crystal structures are both classified as A‐loop: “closed A‐under‐P,” P‐loop: “stretched,” and αC‐helix: “αC‐in” (which is equivalent to HM 12). For the case of sorafenib, a good docking pose was obtained for the “closed type 2” model, which is similar in shape to the “closed A‐under‐P” conformation and three out of the top five selected structures have a closed A‐loop conformation. In the case of axitinib, a matching pose was found in the top five docking solutions but the respective receptor model has a different A‐loop orientation from the crystal structure. Overall, the results imply that the generated ensembles of homology models can be employed for docking calculations, especially when the top poses are found for the same receptor classification state.

## 4 DISCUSSION

### 4.1 Inactive “DFG‐out” states—accessible for every kinase?

One fundamental question of kinome‐wide modeling of DFG‐out conformations is whether every kinase or only a subset of the kinome is capable of adopting the DFG‐out state.^9^ The factors modulating DFG conformations still remain poorly understood. However, several aspects suggest that at least most kinases can adopt every major conformation at a conformational penalty.^5^, ^29^, ^30^ As mentioned earlier, a large ligand‐based profiling study showed that many more kinases compared to those that have a “DFG‐out” structure in the PDB can be targeted by type II inhibitors.^9^ This speaks in favor of most kinases being able to adopt the “DFG‐out” state and might also imply that many of the so far untargeted kinases could potentially be targeted by type II inhibitors. Furthermore, also kinases that were thought to have an inaccessible DFG‐out conformation, such as CDK2,^31^ can bind type II inhibitors and undergo the DFG‐out transition.

Another crucial issue of modeling “DFG‐out” kinases is not only the question of whether kinases generally may adapt a “DFG‐out” state, but also how many different ones. It was shown that for instance ABL1 is able to adapt multiple inactive “DFG‐out” conformations^32^ and that BTK can sample kinome‐wide crystallographically observed states.^33^ Energetic accessibility of the different states might be shifted by multiple factors, such as the phosphorylation state of the A‐loop,^16^ binding of allosteric regulators,^34^ or induction by inhibitors.^5^ This emphasizes the importance and validity of modeling different “DFG‐out” states that have not been observed in crystal structures yet. Most likely not all of the 18 combinations of structural features can be adopted by all kinases but exceptions to that can be accepted while the general idea is valid, especially when it comes to large‐scale modeling and analysis studies.

### 4.2 Modeling conformational plasticity of DFG‐out structures

Previous “DFG‐out” modeling approaches consist of the ALRM,^15^ DOLPHIN,^14^ and DFGmodel^16^ approaches. The ALRM approach is addressing the conformational flexibility of “DFG‐out” states by generating a large number of possible A‐loop conformations and subsequently filtering the generated structures based on the space of the active‐site cleft.^15^ Furthermore, the N‐lobes were rotated about a predefined axis. Thus, ALRM accounts for the variance in the A‐loop and an N‐lobe rotation but not for the other N‐lobe variations, such as different conformations of the P‐loop and αC‐helix. The presented approach accounts for two different P‐loop conformations and three different αC‐helix states but models a lower number of different A‐loop conformations. However, modeling the entire accessible A‐loop conformations might not be necessary for hit identification tasks of small molecules as only the first part, including the DFG motif, and some subsequent residues affect ligand binding; modeling more A‐loop conformations might be of relevance when it comes to the analysis and modeling of protein substrate binding though.^4^

The DFGmodel approach,^16^ as well as DOLPHIN method,^14^ therefore excludes A‐loop residues after the DFG motif from the modeling procedure (ie, DFGmodel: DFG_F_ and the next four residues; DOLPHIN: beyond DFG motif), resulting nevertheless in structures with high practical value for virtual screening. However, also residues after the DFG motif can be involved in forming the binding site (eg, the A‐under‐P structure 3BEA). Furthermore, the presence of the A‐loop might be required for subsequent MD studies.

Similar to the present study, modeling via the DFGmodel approach^16^ is also done based on an ensemble of 18 different template structures. However, the structures were selected based on large variations in the relative position of the N/C lobes and not manually created as done in the present approach. Thus, DFGmodel employs different criteria for the selection/creation of the template structures/modeling of the structural variety of inactive DFG‐out states. Noteworthily, different templates were selected for tyrosine kinases and serine/threonine kinases. Differentiating between different kinase groups might be an interesting improvement of the presented template selection procedure and modeling pipeline, especially when more PDB structures with varying structural elements became available.

## 5 CONCLUSION

The here presented modeling pipeline systematically generates homology models of kinases in the “DFG‐out” state and, thereby, efficiently samples the major conformations of potential value for drug design efforts. The generated structures can not only be employed for docking calculations but can also provide informative insights into selectivity‐determining features, which might be only addressable in scarcely populated conformations.^35^ The basis of the presented pipeline forms a thorough selection of template structures, which were identified after classifying all available kinase structures in the PDB by rules either already described in the literature or newly developed. A systematically diverse set of template structures was identified and employed for the generation of homology models in different “DFG‐out” conformations for all typical kinases in the human kinome. MD simulations further point to the value of the generated ensembles as conformational transitions of the flexible features of the kinase domains (except the αC‐helix) did not occur within the sampled timescale, underlining the potential of the presented ensemble generation pipeline for drug design efforts.

## Supporting information


**Appendix**: Supporting Information
