# Supplementary material for: Modeling conformational flexibility of kinases in inactive states
Source: Proteins. 2019 Jun 17;87(11):943–51. doi: 10.1002/prot.25756 (PMC6852311; doi:10.1002/prot.25756)
Supplement: Supplementary file 1 — Appendix: Supporting Information [file PROT-87-943-s001.pdf]

Supplementary Information

Modeling conformational flexibility of kinases in inactive states

Dominik Schwarz[1,2], Benjamin Merget[1], Charlotte Deane[2], Simone Fulle[1]*

[1] *BioMed X Innovation Center, Im Neuenheimer Feld 515, Heidelberg, Germany*

[2] *University of Oxford, Department of Statistics, 24-29 St Giles', OX1 3LB, United Kingdom*

*Corresponding author: simone.fulle@gmail.com

Table of contents:

Supporting information for docking study

Testing DFG-out model ensembles for docking studies

Supplementary Tables

Table S1: Features of A-loop classifier

Table S2: Features of P-loop classifier

Table S3: Docking set of FDA-approved type II inhibitors for ABL1 and KDR

Table S4: Top docking poses into ABL1

Table S5: Top docking poses into KDR

Supplementary Figures

Figure S1: Distribution of DFG-out structures in the PDB with respect to varying A-loop, P-loop, and αC-helix classes

Figure S2: RMSD values of MD simulations of ABL1 homology models

Figure S3: RMSD values of MD simulations of ABL1 homology models versus 18 classes of kinase conformations

Figure S4: Pairwise relationships five features classifying P-loop conformations

Figure S5: Dependency of RMSD and minimal distances to binding pocket

Testing DFG-out model ensembles for docking studies

Selection of docking test set. PKIDB [1] (http://www.icoa.fr/pkidb/) was used to select the FDA-approved (phase=4) type II (type=2) protein kinase inhibitors of ABL1 and KDR respectively (Table S3).

Receptor and ligand preparation. One PDB structure per ligand and target pair was chosen as a reference (see Table S3), waters were removed from all crystal structures and hydrogens added to all oxygen and nitrogen atoms. All ABL1 complexes (and homology models) were aligned to 2HYY_A and all KDR complexes (and homology models) to 3WZE_A. All receptor structures and ligands were prepared with Open Babel 2.4.1. [2] into pdbqt format. Note that the KDR homology model 9 was not generated by the homology modelling program and that the reference structure of 0LI into ABL1 (PDB 3OXZ) is from *mus musculus.*

Docking calculations. Docking was performed with Smina [3] which is based on AutoDock Vina 1.1.2. [4]. As homology models were generated without the presence of any ligand, the '--flexdist' argument was set to 2.5 Å. Thus, every amino acid's side chain that possessed an atom within 2.5 Å of the respective ligand's coordinates in the crystal structure (set by '--flexdist_ligand' parameter) was treated as flexible. The search space ('--autobox_add') around the respective ligand was set to 5 Å (default: 4 Å). The exhaustiveness parameter's default of 8 was used. A maximum of 9 poses per homology model were output.

Docking analysis. All docking poses of an inhibitor were initially filtered for docking poses within the core binding cavity. This was achieved by measuring distances of the closest inhibitor atom to C_α atoms of the hinge donor (ID 123), αC-helix's Glu (ID 91), and HRD's His (ID 164). To prevent a bias towards poses between the αC-helix and HRD motif, the hinge's distance was double weighted. Dependency of RMSD to the reference crystal structure pose and the measured distance sums are displayed in Figure S5. Visual inspection revealed that poses with an RMSD around 12 Å (and low distance sums) are those inside the binding pocket but in an inverted orientation compared to the crystal structure's binding pose. These poses were kept to resemble a real-world docking scenario. A distance cut-off of 32.5 Å (dashed line) was chosen to exclude poses obviously outside of the binding pocket. This filtering step reduced the number of poses found for STI from 155 to 118, for NIL from 133 to 99, for 0LI from 151 to 119, AXI from 147 to 42 and for BAX from 153 to 107. All remaining docking poses were ordered by ascending energy and the top 5 docking poses of each inhibitor were examined in the presented analysis (see also Table S4 and S5). A 'good matching' pose was defined to have a maximum of 2.2 Å RMSD to the crystal pose.

Supplementary Tables

Table S1: Features of A-loop classifier.

A-loop class	Torsion angle [a]		Distance [b]
	xi DFG{-1, D}	xi DFG{F, G}	C_α-C_α
closed type 2	> 120°	210° ≤ xi < 250°	> 25 Å
open DFG-out	> 120°	-30° ≤ xi < 65°	< 12 Å
closed A-under-P	> 120°	105° ≤ xi < 180°	> 20 Å

[a] Pseudo-torsional angle between four adjacent C_α-atoms: xi DFG{-1, D} with DFG_{-1} (ID 183) and DFG_D (ID 184) being the two central atoms and xi DFG{F, G} with DFG_F (ID 185) and DFG_G (ID 186) being the two central atoms. Note that xi DFG{-1, D} was used by Möbitz [5] to identify the DFG state.

[b] Distance criterion between HRD_{-4} (ID 160) and DFG_{+3} (ID 189). The distance criterion ensures more homogenous clusters compared to using only torsion angles, but excludes a few DFG-out structures.

Compared to the classification scheme by Möbitz [5], 'closed type 2' resembles 'DFG-out type 2', 'open DFG-out' resembles 'DFG-flipped', and 'closed A-under-P' resembles three defined DFG-out 'A-under-P' clusters by Möbitz, respectively.

Table S2: Features of P-loop classifier.

collapsed	stretched
80° < $psi_{\text{G-motif-1}}$	$psi_{\text{G-motif-1}}$ < 50°
40° ≤ $psi_{\text{G-motif+1}}$ ≤ 100°	100° < $psi_{\text{G-motif+1}}$ ≤ 180°
100° ≤ $xi_{\text{G-motif\{+1, +2\}}}$ ≤ 180°	-180° < $xi_{\text{G-motif\{+1, +2\}}}$ < -100°
C_α-C_α ≤ 13.5 Å	C_α-C_α > 13.5 Å

Three out of four of the respective criteria must be fulfilled.

The numbering of *psi* and *xi* refers to the glycine-rich motif (IDs 50-55). $psi_{\text{G-motif-1}}$ and $psi_{\text{G-motif+1}}$ are backbone dihedrals just before and after the GxGxPhiG motif, $xi_{\text{G-motif\{+1, +2\}}}$ is a pseudo-torsional angle between the Cα atoms of the four residues following *Phi* (IDs 55-58), and the Cα-Cα distance is between the *Phi* residue (ID 54) and HRD_{+4} (ID 170).

Table S3: Docking set of FDA-approved type II inhibitors for ABL1 and KDR

INN name	Ligand ID	PDB code	Canonical Smiles
Imatinib	STI	2HYY_A	Cc1ccc(cc1Nc2nccc(n2)c3cccnc3)NC(=O)c4ccc(cc4)CN5CCN(CC5)C
Nilotinib	NIL	3CS9_A	Cc1ccc(cc1Nc2nccc(n2)c3cccnc3)C(=O)Nc4cc(cc(c4)n5cc(nc5)C)C(F)(F)F
Ponatinib	0LI	3OXZ_A	Cc1ccc(cc1C#Cc2cnc3n2nccc3)C(=O)Nc4ccc(c(c4)C(F)(F)F)CN5CCN(CC5)C
Sorafenib	BAX	3WZE_A	CNC(=O)c1cc(ccn1)Oc2ccc(cc2)NC(=O)Nc3ccc(c(c3)C(F)(F)F)Cl
Axitinib	AXI	4AG8_A	CNC(=O)c1ccccc1Sc2ccc3c(c2)[nH]nc3/C=C/c4ccccn4

Table S4: Top docking poses into ABL1.

Ligand	Pose name	Classification of homology model [1]	RMSD [Å] [2]	Affinity [kcal/mol]
	HM_10_pose_1	closed type 2, stretched, αC-in	12.42	-11.4
	HM_ 1_pose_1	closed type 2, collapsed, αC-in *	3.41	-11.2
STI	HM_ 1_pose_2	closed type 2, collapsed, αC-in *	2.12	-11.0
	HM_ 1 pose_3	closed type 2, collapsed, αC-in *	2.12	-11.0
	HM_ 4_pose_1	closed type 2, collapsed, αC-inter	1.52	-11.0
	HM_10_pose_1	closed type 2, stretched, αC-in	11.82	-12.3
	HM_ 1_pose_1	closed type 2, collapsed, αC-in *	3.85	-12.0
NIL	HM_10_pose_3	closed type 2, stretched, αC-in	12.43	-12.0
	HM_10 pose_4	closed type 2, stretched, αC-in	6.40	-12.0
	HM_ 1_pose_2	closed type 2, collapsed, αC-in *	2.12	-11.8
	HM_ 8_pose_1	open DFG-out, collapsed, αC-out	13.11	-11.3
	HM_ 5_pose_1	open DFG-out, collapsed, αC-inter	6.93	-10.9
	HM_ 8_pose_2	open DFG-out, collapsed, αC-out	5.42	-10.9
0LI	HM_18 pose_1	closed A-under-P, stretched, αC-out	7.95	-10.6
	HM_ 5_pose_2	open DFG-out, collapsed, αC-inter	7.06	-10.5
	... (ommiting ranks 6 to 13)			
	HM_16_pose_1	closed type 2, stretched, αC-out	1.12	-9.6

[1] Models that have the same classification with respect to A-loop, P-loop and αC-helix as the crystal structure are marked with an asterix (i.e. A-loop: ‘closed type 2’, P-loop: ‘collapsed’ and αC-helix: ‘αC-in)

[2] To respective crystal pose (all atom RMSD).

Table S5: Top docking poses into KDR.

Ligand	Pose name	Classification of homology model [1]	RMSD [Å] [2]	Affinity [kcal/mol]
AXI	HM_ 4_pose_1	closed type 2, collapsed, αC-inter	6.70	-10.0
	HM_ 1_pose_1	closed type 2, collapsed, αC-in	5.60	-9.4
	HM_ 8_pose_1	open DFG-out, collapsed, αC-out	1.95	-9.4
	HM_ 1 pose_2	closed type 2, collapsed, αC-in	10.35	-8.9
	HM_11_pose_1	open DFG-out, stretched αC-in	10.59	-8.8
BAX	HM_ 8_pose_1	open DFG-out, collapsed, αC-out	6.90	-10.6
	HM_ 1_pose_1	closed type 2, collapsed, αC-in	11.44	-10.3
	HM_18_pose_1	closed A-under-P, stretched, αC-out	5.22	-10.1
	HM_ 1 pose_2	closed type 2, collapsed, αC-in	1.72	-10.0
	HM_ 8_pose_2	open DFG-out, collapsed, αC-out	5.15	-9.9

[1] Models that have the same classification with respect to A-loop, P-loop and αC-helix as the crystal structure are marked with an asterix (i.e. A-loop: 'closed A-under-P', P-loop: 'stretched' and αC-helix: 'αC-in').

[2] To respective crystal pose (all atom RMSD).

Supplementary Figures

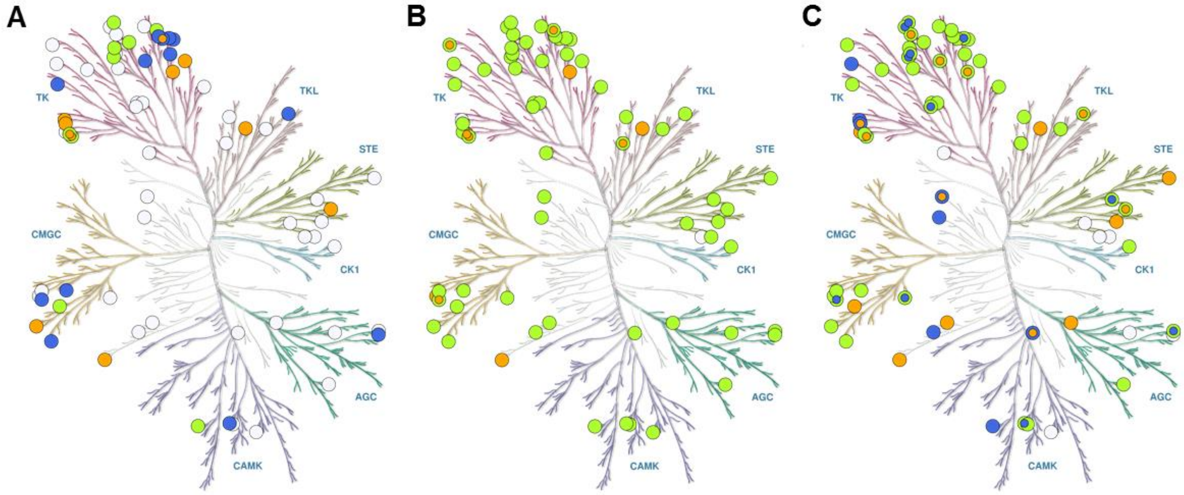

Figure S1: **Distribution of DFG-out structures in PDB with respect to varying A-loop, P-Loop, and αC-helix classes.**

Coloured are kinases that have at least one DFG-out structure in the respective class, while those without assigned classes, either due to structural incompleteness (i.e. in the case of the P-loop) or due to differing geometrical criteria, are coloured in white. All kinome tree figures were generated via KinMap (www.kinhub.org/kinmap/).[6]

(A) Distribution of A-loop classes *'closed type 2'* (green), *'open DFG-out'* (orange) or *'closed A-under-P'* (blue). 'Closed type 2' conformations are only detected so far for the TK, CAMK, and CMGC groups. However, a profiling study of type II inhibitors [7] showed that these inhibitors can target more than 200 kinases, suggesting that most kinases can form a 'closed type 2' or 'closed A-under-P' A-loop. Thus, we decided to include both conformational classes as templates of the modelling procedure of the entire kinome. 'Open DFG-out' conformations are relatively even distributed amongst the kinome and only two kinase groups lack an existing PDB structure of this conformation. Hence, a kinome-wide distribution can be also assumed.

(B) Distribution of P-loop classes *'collapsed'* (orange) and *'stretched'* (green). The 'stretched' P-loop conformation occurs in every kinase group, whereas the number of 'collapsed' conformations in DFG-out structures are overall lower. Since collapsed P-loop conformations in DFG-in structures occur in every kinase group (data not shown), we assumed this structural class to be potentially also sampled in the entire kinome in the DFG-out state.

(C) Distribution of αC-helix classes *'aC-out'* (orange), *'aC-inter'* (blue), and *'aC-in'* (green). The classification was obtained by employing the Brooijmans αC-helix classifier.[8] All three conformations are present in most kinase groups. Hence, a kinome-wide distribution of all three αC-helix classes can be assumed and all three classes were included as templates of the modelling procedure.

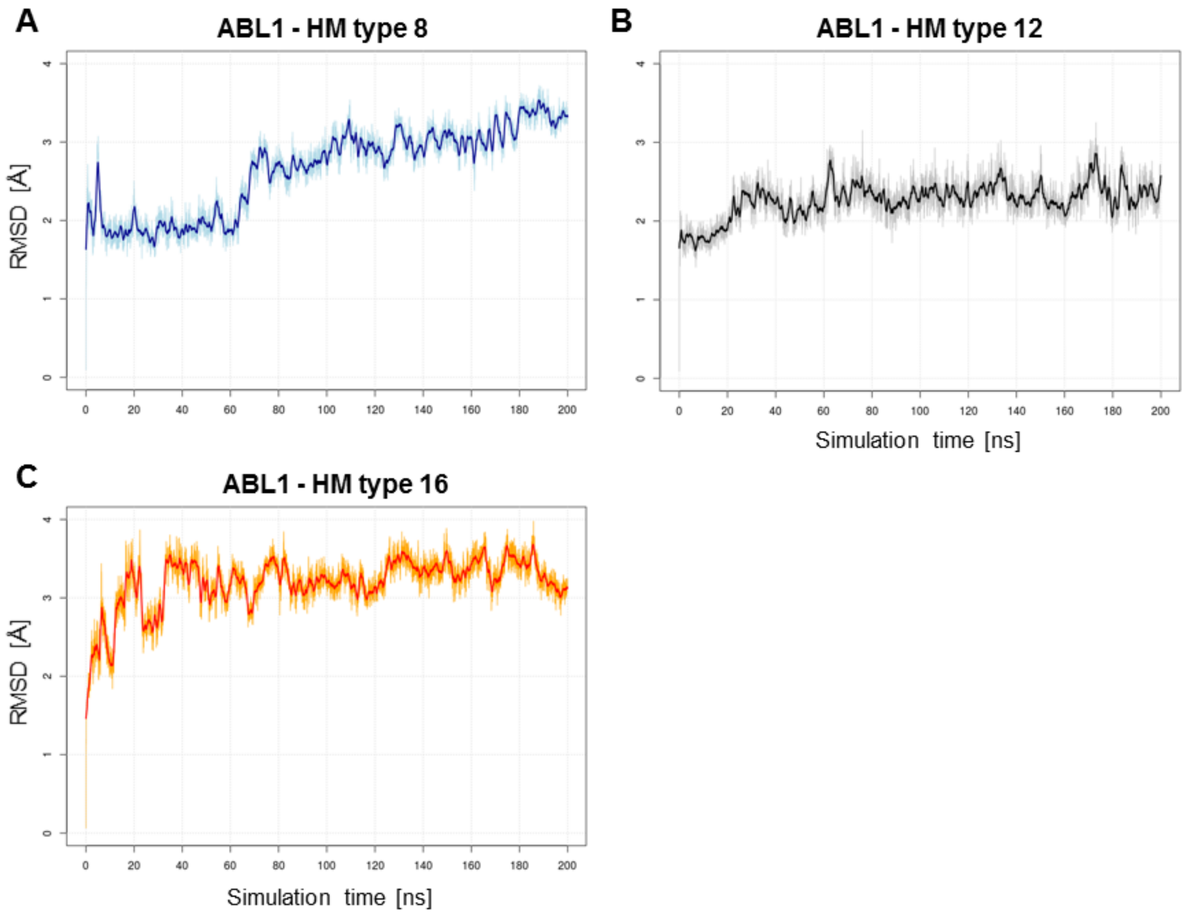

Figure S2: **RMSD values of MD simulations of ABL1 homology models.** RMSD values were calculated for the entire structure considering only the heavy backbone atoms and indicate stable trajectories without large conformational changes.

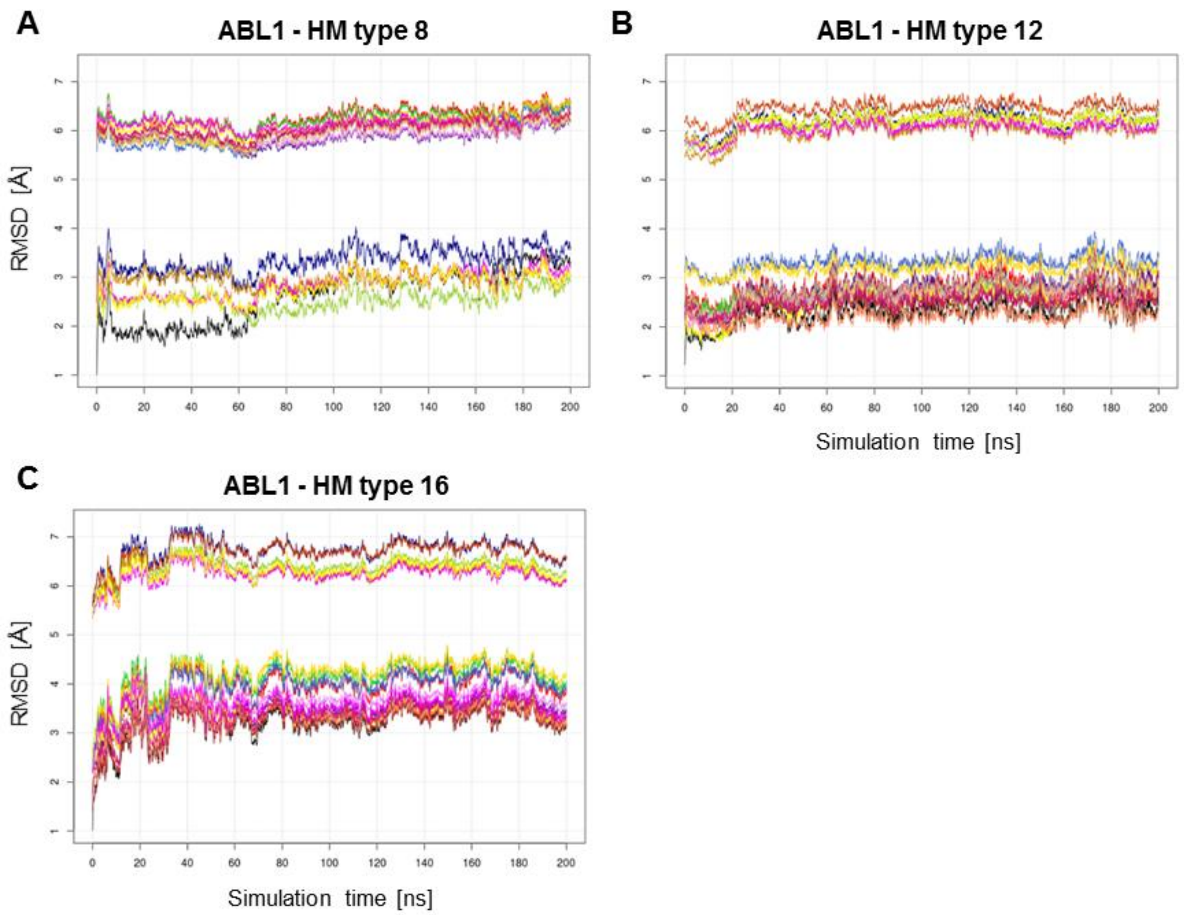

Figure S3: **RMSD values of MD simulations of ABL1 homology models versus 18 classes of kinase conformations.** RMSD values are grouped together with respect to structures with an A-loop in '*open DFG-out'* conformation (#6 starting structures: HM 2: dark blue; HM 5: olive green; HM 8: orange red; HM 11: dark orange; HM 14: pink; HM 17: khaki) as well as with respect to structures with closed A-loop conformations '*closed type 2'* (#6 starting structures: HM 1: light red, HM 4: purple, HM 7: blue; HM 10: yellow; HM 13: brown; HM 16: light brown) and '*closed A-under-P'* (#6 structures: HM 3: green; HM 6: violet; HM 9: gold; HM 12: magenta; HM 15: salmon; HM 18: dark red). The RMSD values indicate that they are mainly influenced by the differing A-loop conformations and that no major structural changes occur for this loop within MD trajectories of 200 ns length. Black curves in all three plots correspond to RMSD values of the respective MD trajectory in comparison to the starting structure.

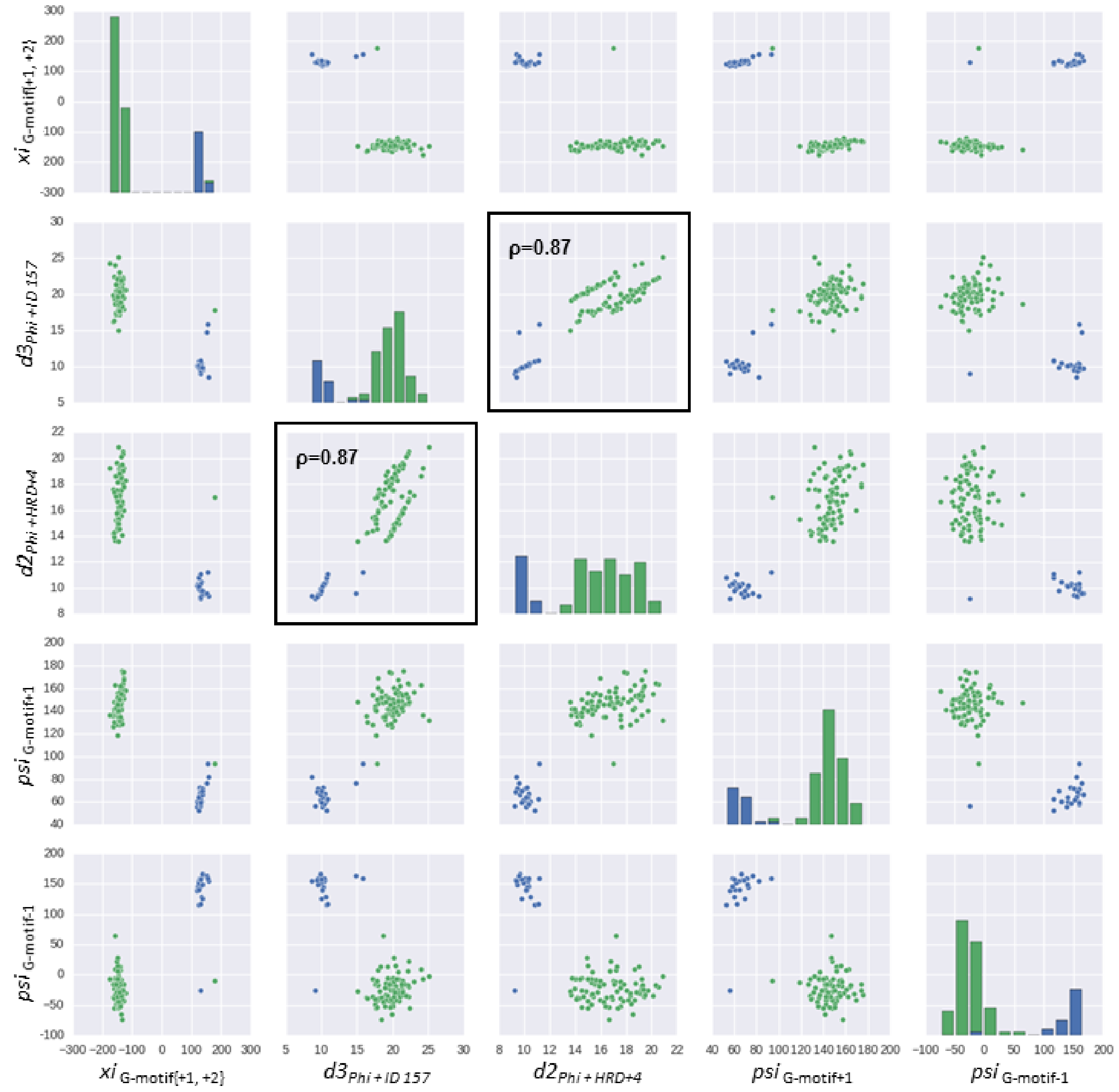

Figure S4: **Pairwise relationships of five features classifying P-loop conformations.** The P-loop conformations can be mainly differentiated into '*stretched'* (green) and '*collapsed'* (blue). The analysis of the derived feature importance of a trained random forest classifier revealed the five plotted features in the figure to be of high potential value for P-loop classification. The feature d3, describing C_α-C_α distances, was subsequently removed due to the high correlation with feature d2. The initial 40 structural features of the P-loop residues included: *phi* and *psi* backbone dihedrals of 13 P-loop residues (IDs 47-59), 10 pseudo-torsional angles of the same 13 residues and 4 C_α-C_α distances between either the first Gly of the GxGxPhiG motif (ID 50) or the *Phi* (Tyr/Phe) residue of the same motif (ID 54) and either the HRD_{+4} residue (ID 170) or the third residue following the hinge's end (ID 127).

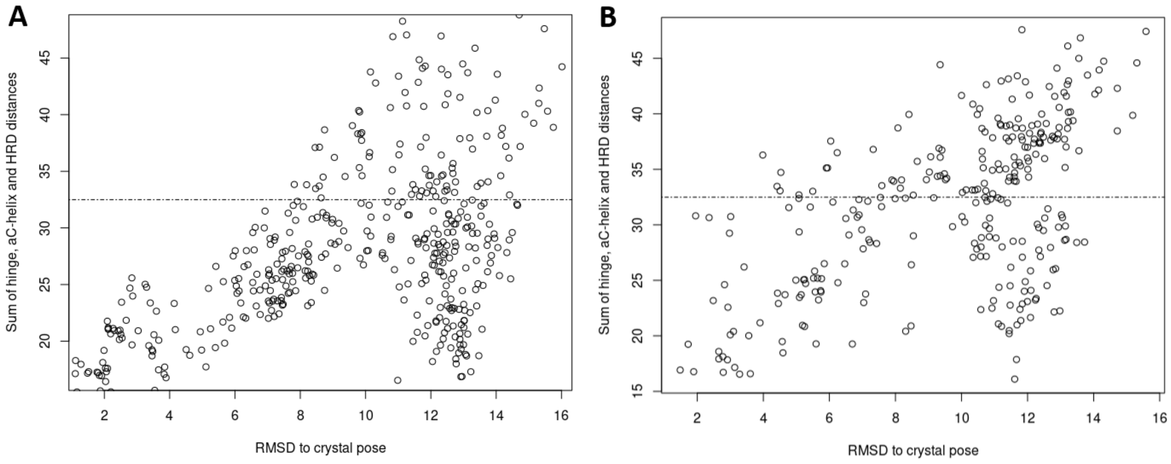

Figure S5: Dependency of RMSD and minimal distances to binding pocket.
Docking poses of (A) STI, NIL and 0LI into ABL1 and (B) of AXI and BAX into KDR. Filtering threshold is shown as dashed line (32.5 Å).

References

1. Carles, F., Bourg, S., Meyer, C., and Bonnet, P. (2018) PKIDB: A curated, annotated and updated database of protein kinase inhibitors in clinical trials. *Molecules*, **23** (4), E908.
2. O'Boyle, N., Banck, M., James, C., Morley, C., and Vandermeersch, T Hutchison, G. (2011) Open Babel: An open chemical toolbox. *J. Cheminform.*, **3**, 33.
3. Koes, D., Baumgartner, M., and Camacho, C. (2013) Lessons learned in empirical scoring with smina from the CSAR 2011 benchmarking exercise. *J. Chem. Inf. Model.*, **53** (8), 1893–1904.
4. Trott, O., and Olson, A. (2010) AutoDock Vina: improving the speed and accuracy of docking with a new scoring function, efficient optimization, and multithreading. *J. Comput. Chem.*, **31** (2), 455–461.
5. Möbitz, H. (2015) The ABC of protein kinase conformations. *Biochim. Biophys. Acta*, **1854** (10), 1555–1566.
6. Eid, S., Turk, S., Volkamer, A., Rippmann, F., and Fulle, S. (2017) KinMap: a web-based tool for interactive navigation through human kinome data. *BMC Bioinformatics*, **18**, 16.
7. Zhao, Z., Wu, H., Wang, L., Liu, Y., Knapp, S., Liu, Q., and Gray, N.S. (2014) Exploration of type II binding mode: a privileged approach for kinase inhibitor focused drug discovery? *ACS Chem Biol*, **9** (6), 1230–1241.
8. Brooijmans, N., Chang, Y.-W., Mobilio, D., Denny, R.A., and Humblet, C. (2010) An enriched structural kinase database to enable kinome-wide structure-based analyses and drug discovery. *Protein Sci*, **19** (4), 763–774.
